# Genomic heterogeneity at baseline is associated with T790M resistance mutations in *EGFR*‐mutated lung cancer treated with the first‐/second‐generation tyrosine kinase inhibitors

**DOI:** 10.1002/cjp2.354

**Published:** 2024-01-17

**Authors:** Michael Menzel, Martina Kirchner, Klaus Kluck, Markus Ball, Susanne Beck, Michael Allgäuer, Christin Assmann, Johannes Schnorbach, Anna‐Lena Volckmar, Timothy Kwang Yong Tay, Hannah Goldschmid, Daniel SW Tan, Michael Thomas, Daniel Kazdal, Jan Budczies, Albrecht Stenzinger, Petros Christopoulos

**Affiliations:** ^1^ Institute of Pathology Heidelberg University Hospital Heidelberg Germany; ^2^ Translational Lung Research Center (TLRC) Heidelberg Member of the German Center for Lung Research (DZL) Heidelberg Germany; ^3^ Department of Thoracic Oncology Thoraxklinik and National Center for Tumor Diseases at Heidelberg University Hospital Heidelberg Germany; ^4^ Department of Anatomical Pathology Singapore General Hospital Singapore; ^5^ Department of Clinical Trials and Epidemiological Sciences National Cancer Centre Singapore

**Keywords:** non‐small cell lung cancer (NSCLC), epidermal growth factor receptor (EGFR), T790M mutation, whole exome sequencing (WES), tyrosine kinase inhibitor, tumor heterogeneity

## Abstract

This study analyzed whether extended molecular profiling can predict the development of epidermal growth factor receptor (*EGFR*) gene T790M mutation, which is the most frequent resistance alteration in non‐small cell lung cancer (NSCLC) after treatment with the first‐/second‐generation (1G/2G) EGFR inhibitors (tyrosine kinase inhibitors [TKIs]), but only weakly associated with clinical characteristics. Whole exome sequencing (WES) was performed on pretreatment tumor tissue with matched normal samples from NSCLC patients with (*n* = 25, detected in tissue or blood rebiopsies) or without (*n* = 14, negative tissue rebiopsies only) subsequent *EGFR* p.T790M mutation after treatment with 1G/2G EGFR TKI. Several complex genetic biomarkers were assessed using bioinformatic methods. After treatment with first‐line afatinib (44%) or erlotinib/gefitinib (56%), median progression‐free survival and overall survival were 12.1 and 33.7 months, respectively. Clinical and tumor genetic characteristics, including age (median, 66 years), sex (74% female), smoking (69% never/light smokers), *EGFR* mutation type (72% exon 19 deletions), and *TP53* mutations (41%) were not significantly associated with T790M mutation (*p* > 0.05). By contrast, complex biomarkers including tumor mutational burden, the clock‐like mutation signature SBS1 + 5, tumor ploidy, and markers of subclonality including mutant‐allele tumor heterogeneity, subclonal copy number changes, and median tumor‐adjusted variant allele frequency were significantly higher at baseline in tumors with subsequent T790M mutation (all *p* < 0.05). Each marker alone could predict subsequent development of T790M with an area under the curve (AUC) of 0.72–0.77, but the small number of cases did not allow confirmation of better performance for biomarker combinations in leave‐one‐out cross‐validated logistic regression (AUC 0.69, 95% confidence interval: 0.50–0.87). Extended molecular profiling with WES at initial diagnosis reveals several complex biomarkers associated with subsequent development of T790M resistance mutation in NSCLC patients receiving first‐/second‐generation TKIs as the first‐line therapy. Larger prospective studies will be necessary to define a forecasting model.

## Introduction

Epidermal growth factor receptor (EGFR) tyrosine kinase inhibitors (TKIs) have revolutionized the treatment of advanced *EGFR*‐mutated non‐small cell lung cancer (NSCLC) with prolonged overall survival (OS) compared with platinum‐based doublet chemotherapy in several phase III trials [1, 2, 3, 4, 5]. Head‐to‐head comparative studies have shown that the third‐generation (3G) TKI osimertinib is superior compared with the first‐generation TKIs [5] and is now the preferred the first‐line TKI in these patients according to most treatment guidelines [6].

*EGFR* p.T790M is the most common resistance mutation arising after treatment with the first‐ and second‐generation TKIs, such as erlotinib, gefitinib, and afatinib, occurring in 50–60% of patients. It has major therapeutic importance, because it confers sensitivity to next‐line osimertinib [7, 8]. Interestingly, real‐world analyses have shown an OS of 36–41 months for patients receiving earlier generation EGFR TKI followed by osimertinib [9, 10], which is at least as good as that of upfront osimertinib in the FLAURA study [5]. However, the practicability of a sequential TKI strategy is limited by the fact that the development of the T790M resistance mutation and eligibility for next‐line osimertinib cannot be reliably predicted based on clinical features or basic molecular tumor characteristics before the start of the first‐line treatment, because the corresponding correlations are weak [11]. The aim of this exploratory retrospective analysis was to explore whether deep molecular profiling using whole exome sequencing (WES) of tumor tissue at initial diagnosis could facilitate a more reliable prediction of the subsequent development of *EGFR* p.T790M for patients with *EGFR* mutated NSCLC receiving first‐/second‐generation EGFR TKI as the first‐line therapy.

## Patients and methods

### Patient population

Included in this study were patients with *EGFR* mutated NSCLC who (1) received first‐/second‐generation TKI as the first‐line treatment in the Thoraxklinik Heidelberg between 2014 and 2018; (2) had known *EGFR* p.T790M status at the time of TKI resistance, either T790M‐positive (‘T790Mpos’), based on a positive tissue or liquid rebiopsy, or T790M‐negative (‘T790Mneg’), based on a negative tissue biopsy only, to minimize the effect of false negative results known to occur in up to 1/3 of cases undergoing circulation tumor DNA only testing [12]; and (3) had available tumor tissue and normal (i.e. germline) DNA samples for WES. Among 79 primarily identified patients, 39 could be analyzed (49%). WES could not be performed in the remaining cases because of insufficient quantity or quality of tumor DNA or because normal control samples were not available. Ethical approval was provided by the ethics committee of Heidelberg University (S‐935/2021).

### Histopathological methods

Microscopic analysis of the following parameters was performed by two experienced pathologists who were blinded to the genetic results: predominant growth pattern/grading according to the current WHO classification of thoracic tumors, nuclear cytology, stroma, inflammation, immunohistochemical staining of TTF1 and Ki‐67 (if available), and necrosis. Inflammation in most cases was lymphoplasmacytic. A qualitative statement was rendered based on light microscopic impression. A mild inflammatory infiltrate was defined as scattered and rarely aggregated lymphocytes and plasma cells. A marked inflammatory infiltrate was defined by the extensive presence of lymphocytes and plasma cells constituting a prominent feature of the tumor. Cases falling between mild and marked were classified as moderate. Results were subsequently analyzed using the Fisher exact test with Bonferroni correction for multiple testing.

### Molecular methods

WES was performed using 100 ng DNA with the Twist Exome 2.0 plus Comprehensive Exome Spike‐in (Illumina, San Diego, CA, USA) on a NovaSeq sequencer (Illumina) with an average coverage of 170×. Somatic variants were kept after WES of matched normal blood or tissue samples from the same patients using 100 ng DNA with the same method and an average coverage of 50×.

### Statistical analysis and bioinformatics

Alignment was performed using the Illumina DRAGEN Bio‐IT platform version 4.0.3 with the genome assembly GRCh37. Somatic variants were filtered with a PASS filter, including exonic and splice regions with variant allele frequency ≥5% and coverage ≥100 reads.

Tumor mutational burden (TMB) of mutations per mega‐base (MB) was calculated as the count of mutations using the sum of missense and synonymous variants divided by the target region length, defined by the count of MB covered with at least 100 reads.

The classification of mutations belonging to established mutational (single‐base substitution [SBS]) signatures and the enrichment analysis of various pathways in the detected mutations according to the Molecular Signatures Database were conducted as published [13].

Samples were subject to segmented copy‐number (CN) evaluation and subclonality analysis using Sclust [14] excluding sex chromosomes and using the default software parameters. Somatic mutations and CN changes were classified in clonal (*p* ≥ 0.05) and subclonal (*p* < 0.05). Subclonal TMB (scTMB) and fraction of subclonal CN changes were calculated based on this classification.

Genome‐wide copy‐number alterations were also calculated using Sequenza [15] using the default parameters. Subsequently, loss of heterozygosity (LOH), large‐scale transitions, telomeric allelic imbalance, and homologous recombination deficiency (HRD) scores for each sample were calculated using scarHRD [16]. The HRDsum score solution was chosen based on the histopathologically determined tumor purity by selecting the best solution for the given purity and ploidy as described previously [17]. Thereby, the corrected tumor ploidy was also determined for the given histopathological tumor purity. Chromosome arm losses were defined as published [18]: in short, we searched for coherent losses of both alleles in the CN identified by Sequenza that span at least 80% of the chromosome arms.

Gene amplifications and deletions were calculated based on the total CN from Sequenza corrected for the histopathologically determined tumor purity, as described previously. A gain of two or more copies from the predominant CN was regarded as amplification, whereas a loss of two or more copies was regarded as deletion.

Tumor‐adjusted variant allele frequency (TVAF) was calculated for each mutation using the following formula:

$$\text{TVAF}=\frac{h}{q}\mathrm{VAF}.$$

The factor *h* was set to 0.5 if heterozygosity was lost in the region of the mutation and set to 1 if not. The factor *q* is a corrected variant of the pathological tumor purity *p* taking into account copy number CN ≠ 2 in the region of the mutation:

$$q=\frac{\mathrm{CN}}{\mathrm{CN}\times p+2\left(1-p\right)}p.$$

A large discrepancy between bioinformatically and histopathologically determined tumor purity was observed (median bioinformatic estimation: 30% versus 50% histopathologically, median delta 16%); therefore, the latter was used in this study.

Mutant allele tumor heterogeneity (MATH) score was calculated as published [19, 20]. Read counts supporting the resistance mutation were identified using bam‐readcount [21].

Statistical analyses and figure generation were performed using Python 3.10.10 with scipy [22], numpy [23], Matplotlib [24], seaborn [25], and Pandas [26]. Significance between groups for each biomarker was evaluated using the Wilcoxon rank‐sum tests for continuous variables and using Fisher tests for binary variables. *p* values for the association of biomarkers with T790M status were corrected using the Benjamini–Hochberg method and evaluated using a false discovery rate (FDR) of 10%. Correlations were calculated according to Pearson. Combinations of biomarkers were evaluated using the logistic regression from scipy [22]. Receiver operating characteristic (ROC) curves were created using sklearn and the R package pROC [27] for area under the curve (AUC) intervals. Survival analyses based on Cox proportional‐hazard models were performed using the R packages survival [28] and survminer [29] with the Wald test for significance testing.

## Results

### Patient characteristics

**Figure 1:**
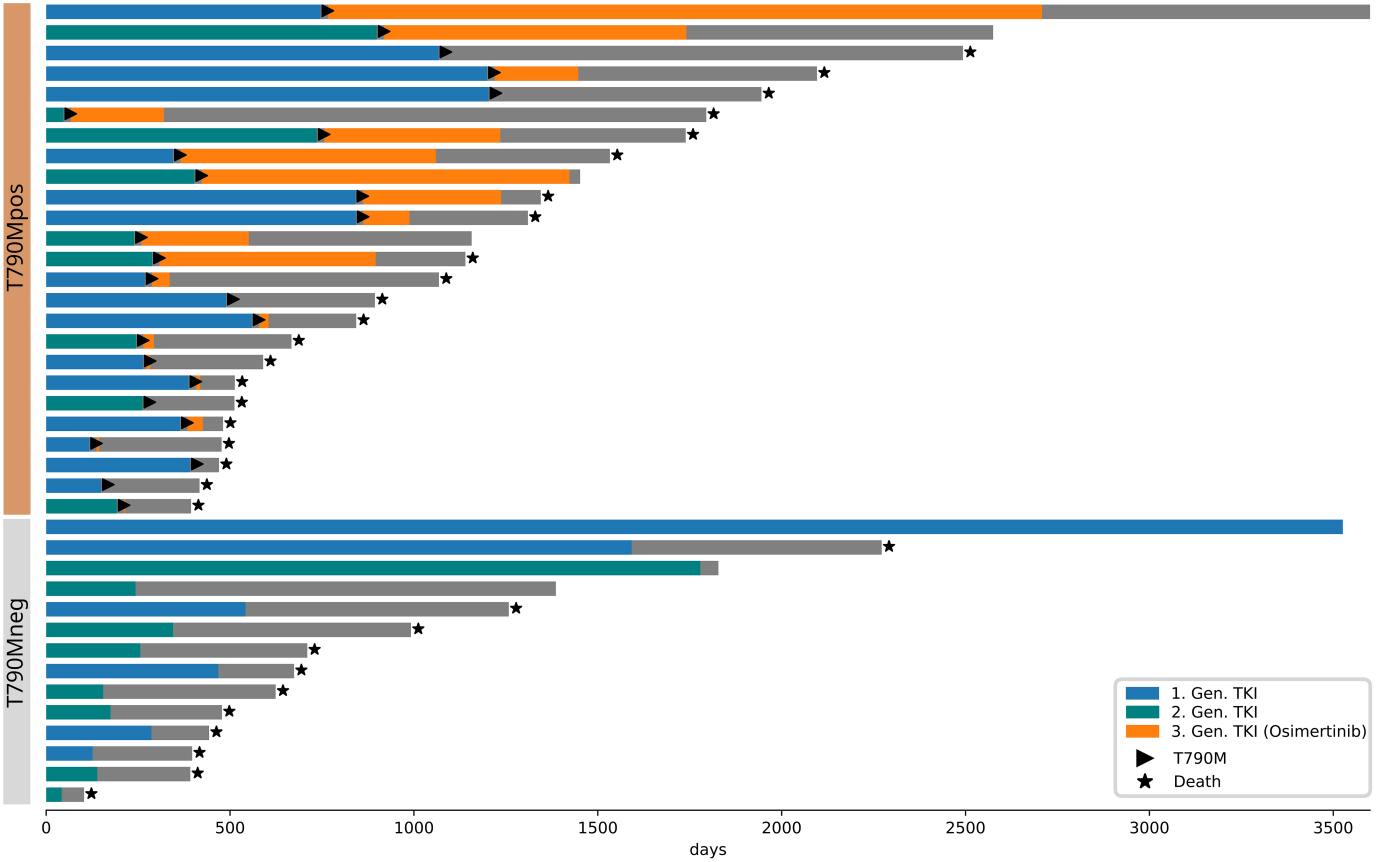
Swimmer's plot of patient cohort. Patients are separated by their individual resistance mechanism. As part of standard of care practices, third‐generation *EGFR* inhibitor osimertinib was only given to patients with detection of the *EGFR* p.T790M resistance mutation.

Overall, 39 (49%) patients with NSCLC who fulfilled the study criteria could be identified, among 79 potentially suitable patients, as tumor and normal tissue WES were not available for 40 cases due to insufficient quantity or quality of tumor DNA, or because no normal control samples were available. The clinical characteristics of patients analyzed with WES are given in Table 1, whereas the characteristics of all 79 patients are given in supplementary material, Table S1. Median age was 66 years with a predominance of female (74%) never/light‐smokers (<10 pack‐years, 69%). Median progression‐free survival (PFS) of the first‐/second‐generation TKI treatment was 365 days (range, 62–3,546 days). The *EGFR* p.T790M resistance mutation was detected in 25 of 39 patients (64%) in the cohort. The third‐generation TKI osimertinib was given as the second‐line treatment to 19 patients (49%) and led to an additional median PFS of 248 days (range, 9–1,962 days). Individual progressions are shown in Figure 1. None of the clinical characteristics or basic molecular features (*EGFR* mutation type and presence of *TP53* mutations) at baseline was significantly associated with the subsequent development of EGFR p.T790M in our cohort (Table 1 and Figure 1).

**Table 1:** Characteristics of patients with WES of tumor and normal tissue.

All study patients	WES patients (*n* = 39)	T790Mpos (*n* = 25)	T790Mneg (*n* = 14)	*p* value
Age, median (IQR)	66 (56–75)	66 (54–75)	62 (58–75)	0.86
Sex, female, *n* (%)	29 (74)	19 (76)	10 (71)	0.75
ECOG PS 0/1, *n*	20/19	14/11	6/8	0.43
Never/light smokers, *n* (%)	27 (69)	19 (76)	8 (57)	0.22
Stage IV at initial diagnosis, *n* (%)	29 (74)	17 (68)	12 (86)	0.22
Brain metastases, *n* (%)	10 (26)	6 (24)	4 (29)	0.74
*EGFR*mut: del19/L858R/other, *n*	28/9/2	21/3/1	7/6/1	0.07
*TP53* mutated/wild type	16/23	12/13	9/5	0.25
PFS of 1L EGFR TKI, median (IQR), mo	12.1 (8.6–25.5)	13.6 (9.4–25.5)	9.2 (5.8–18.7)	0.70
OS, median (IQR)	33.7 (16.7–65.5)	38.7 (17.8–60.5)	23.3 (15.4–76.4)	0.69

1L, first line; ECOG PS, Eastern Cooperative Oncology Group performance status; IQR, interquartile range; mo, months; *n*, number.

### Histopathological features

Nine predefined histopathological features were evaluated by two experienced pathologists (supplementary material, Table S2). The association of the histopathological features with the resistance mechanisms was not significant after correcting for testing multiplicity (FDR = 5%). Nonsignificant higher immune infiltration was observed when the resistance was mediated by T790M mutation compared with other resistance mechanisms (moderate‐to‐strong lymphocyte infiltration in 73% versus 30% of cases, *p* = 0.0069).

### Molecular features

As the samples were taken before the development of the resistance mutation, we expected an absence of reads supporting the *EGFR* p.T790M mutation. For confirmation, we interrogated each sample for the occurrence of *EGFR* c.2369C>T. While there were some reads observed with this mutation in 15 samples from both the T790Mpos and T790Mneg groups, the VAF was below 1% in all cases, and no significant difference was found between the T790Mpos and T790Mneg groups (mean 0.17% versus 0.18%, *p* = 0.85).

**Figure 2:**
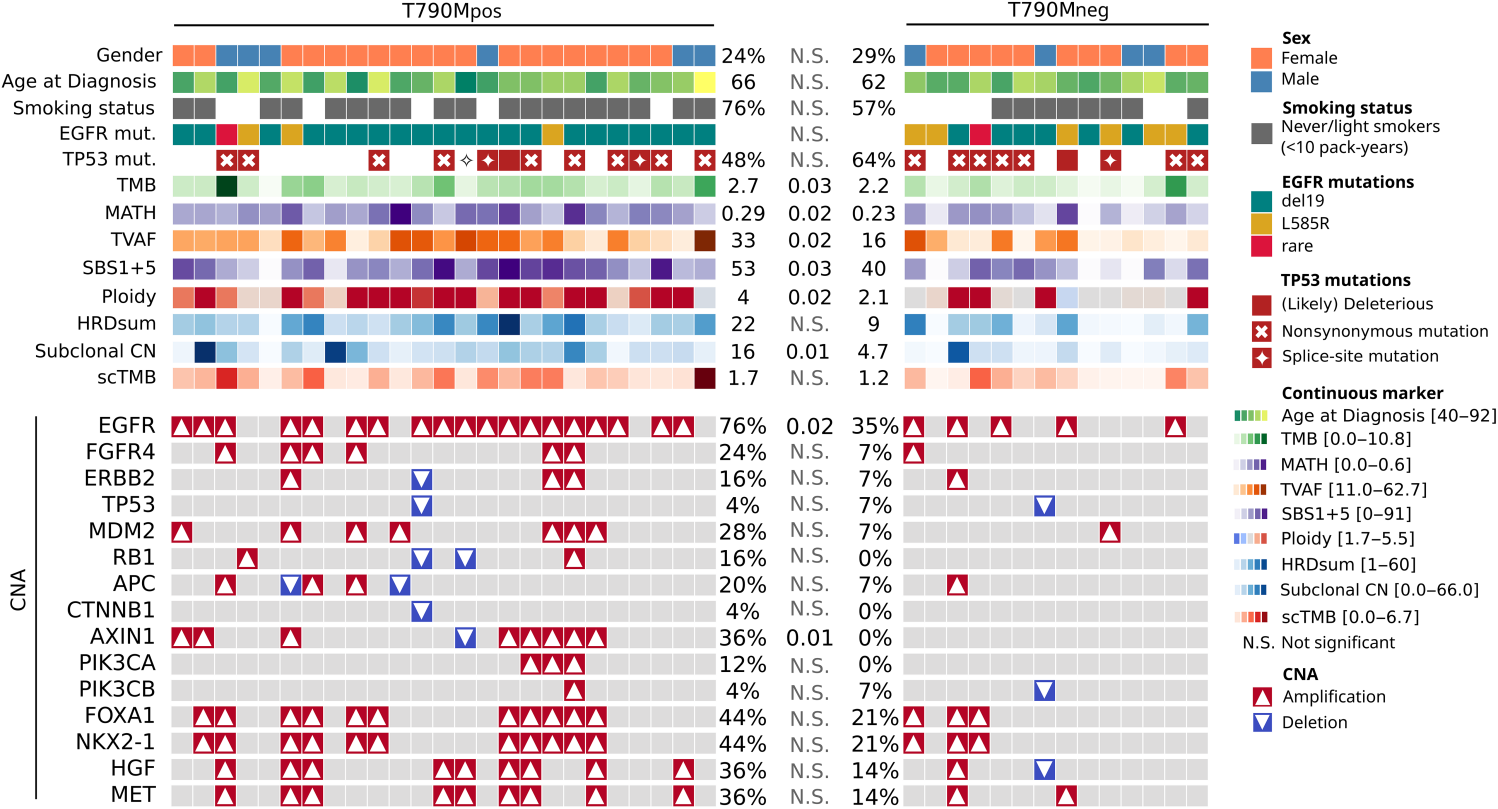
Clinical and molecular patient characteristics. Each column corresponds to an individual patient. Statistical comparisons of patient characteristics between the T790Mpos and T790Mneg groups were performed using the Wilcoxon rank‐sums and Fisher exact tests, with the statistical significance indicated in the figure.

To identify biomarkers that predict the development of T790M resistance mutations, we evaluated a multitude of complex biomarkers based on the WES data of both patient groups (Figure 2). NSCLC is known to harbor *TP53* comutations [30], which we identified belonging to pathogenic and likely pathogenic categories in 21 cases (54% of the cohort patients, 48% in T790Mpos, and 64% in T790Mneg), yet they did not display a significant difference between both groups (Figure 2 and supplementary material, Table S2). While there were several other mutations present in both groups, we did not identify statistically significant differences for any mutation between T790Mpos and T790Mneg cases (supplementary material, Table S3). However, considering mutations collectively as a whole with TMB as a surrogate measure, there was a significant difference, with the T790Mpos cases showing a median TMB of 2.7 versus 2.2 in the T790Mneg cases (*p* = 0.03, Figure 2), indicating that a higher mutational burden increases the likelihood for the subsequent development of the *EGFR* p.T790M resistance mutation. Further, the scTMB was numerically higher in the T790Mpos group (median 1.7 versus 1.2), but the result was not statistically significant (*p* = 0.07).

Furthermore, we evaluated different complex biomarkers referring to tumor heterogeneity. The MATH score, a recently introduced measure of tumor heterogeneity [19], revealed a significant difference between both groups with the T790Mpos group expressing significantly higher tumor heterogeneity (median 0.29 versus 0.23, *p* = 0.03). Besides, we recalculated the MATH scores from a retrospective study [18] comparing tissue samples of rebiopsies at the time of TKI failure for tumors with the T790M resistance mutation against other resistance mutations and found no significant difference between T790Mpos and T790Mneg (0.33, 0.35, *p* = 0.97, supplementary material, Table S4). The median VAF of somatic mutations acts as another surrogate marker for tumor heterogeneity. To subtract the effects of tumor purity and LOH at mutation sites, we calculated a TVAF and observed a significantly higher median TVAF of 33 in the T790Mpos group compared with 16 in the T790Mneg group (*p* = 0.02).

The evaluation of SBS mutational signatures showed the occurrence of multiple signatures (SBS2, SBS1 + 5, SBS4, SBS13, SBS30, SBS92, and SBS96; see supplementary material, Table S2) in the study cohort, but only a single signature (SBS1 + 5, clock‐like mutational process) was significantly different between both groups occurring with a median percentage of 53 in the T790Mpos group versus 40 in T790Mneg (*p* = 0.03, Figure 2).

In 15 (38%) cases, we observed discrepancies larger than 30% between the bioinformatically and histopathologically determined tumor purity (supplementary material, Table S2). As the bioinformatically determined tumor ploidy using Sequenza is set as a function of tumor purity, we, therefore, adjusted the ploidy estimation to adhere to the histopathologic tumor purity. In the resulting ploidy estimates, we observed a significant difference between the groups (Figure 2) with a median ploidy of 4 in the T790Mpos group versus 2.1 in T790Mneg (*p* = 0.02). The majority (80%) of tumors in the T790Mpos group showed a genome duplication compared with only four (29%) tumors in the T790Mneg group (*p* = 0.0002).

Genome instability (GI) was measured using the HRDsum score, which was also adjusted for the histopathologically determined tumor purity as described previously [17]. GI in the T790Mpos group was numerically higher with a median HRDsum score of 22 versus 9 in the T790Mneg group, yet not statistically significant (*p* = 0.059). We further evaluated the CN changes in both groups as the fraction of regions altered, which did not differ between the groups (*p* = 0.46, supplementary material, Table S2). However, the fraction of subclonal CN did reveal a significantly higher occurrence of subclonal CN events in T790Mpos mutated cases with a median of 16% in the T790Mpos group compared with 4.7% in T790Mneg cases (*p* = 0.01).

We evaluated LOH of chromosome arms in both sample groups and observed several LOH in different samples (supplementary material, Table S6). However, none were statistically significant, with the best being chr21 p with *p* = 0.16. Furthermore, only a single deep loss of a chromosome arm was observed in the cohort (chr21 q).

### Prediction of T790M development

**Figure 3:**
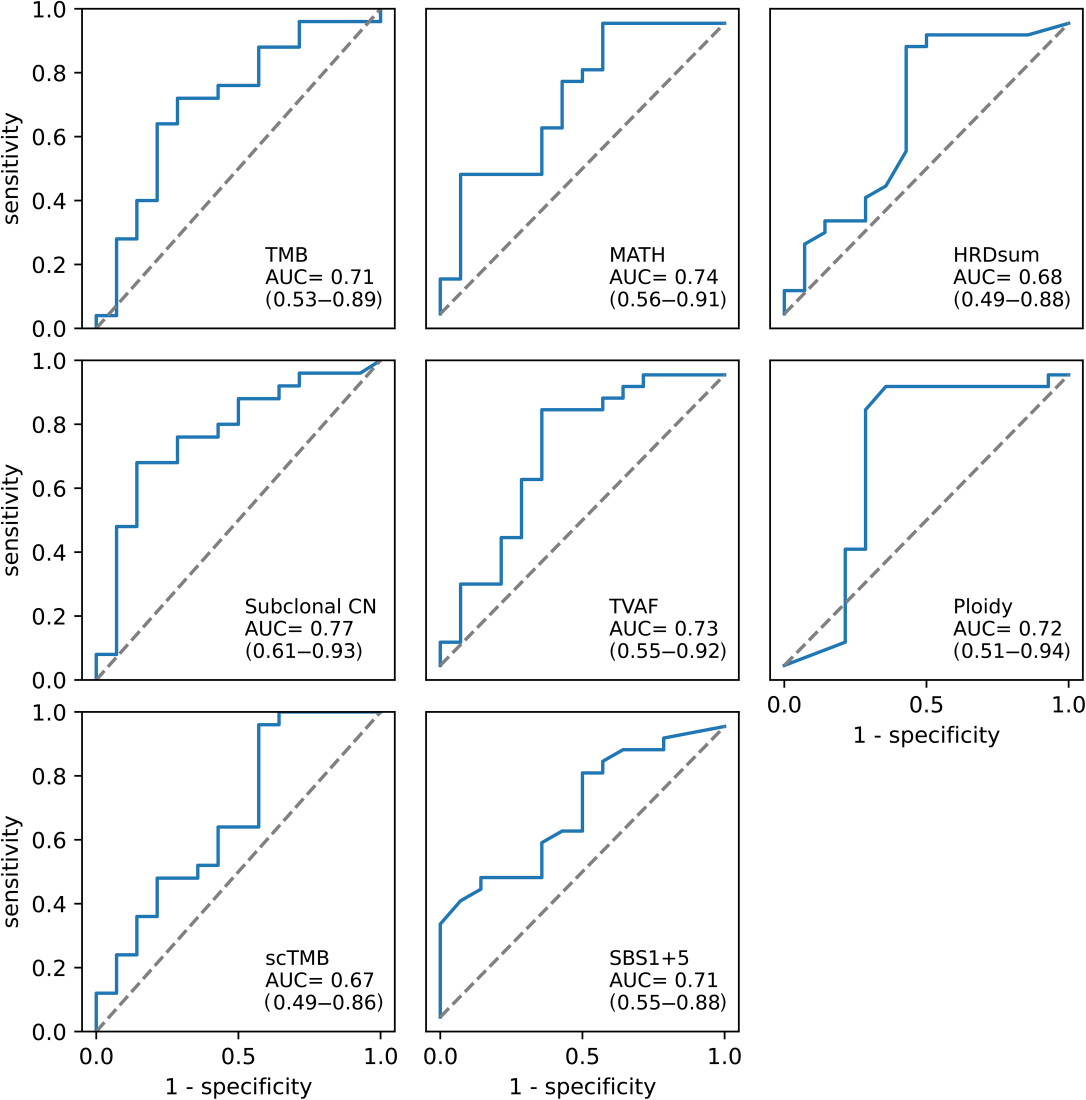
The ability of individual genetic biomarkers to predict the subsequent development of *EGFR* p.T790M mutations. Area under the ROC curve (AUC) for individual genetic biomarkers with statistically significant differences between T790Mpos and T790Mneg patients.

To build a prediction model of T790M mutations, the discriminatory properties of the best‐performing biomarkers were assessed. The fraction of subclonal CN did reveal the highest discriminatory power with an AUC ROC of 0.77, followed by MATH (0.74), TVAF (0.73), and the adjusted ploidy (0.72) (Figure 3).

As no single discriminator with clinically satisfactory performance was identified, we continued by building a model using combinations of biomarkers. To exclude biomarkers reflecting the same biological characteristics, we calculated the pairwise correlations between each marker. The highest correlations were observed between TMB and scTMB (*R* = 0.79), between SBS1 + 5 and HRDsum (*R* = 0.67), as well as between TVAF and HRDsum (*R* = 0.56), whereas no other correlation exceeded *R* > 0.5 (supplementary material, Table S7). We, therefore, excluded the combination of TMB and scTMB, as well as HRDsum with SBS1 + 5 and TVAF from the combined analysis.

We evaluated the performance of a logistic regression model combining the highly differential features (TMB, fraction of subclonal CN, MATH, TVAF, SBS1 + 5, and ploidy) and validated our approach with leave‐one‐out cross‐validation (LOOC). The model achieved an AUC of 0.69 (CI 0.5–0.87) for the prediction of T790M mutations in our cohort, which did not exceed the previously found AUCs of the individual parameters. This was probably due to the relatively small number of cases, which caused unstable behavior of the utilized algorithms. Using LOOC, we were able to validate the applicability of the logistic regression model, yet were not able to define exact weights for the parameters used.

### Survival analysis

The association of complex genetic biomarkers with PFS and OS measured from the application of the first‐line therapy was weak. The only significant result was a slightly increased hazard ratio (HR) for higher TVAF (HR = 1.06, *p* = 0.032, supplementary material, Figure S1).

## Discussion

Considering the long survival of patients with *EGFR* mutated NSCLC who received sequential first/second generation followed by the third‐generation TKIs due to the development of T790M, along with increasing use of large next generation sequencing (NGS) panels and WES as routine molecular workup at least in the larger academic institutions in the foreseeable future [31], this study analyzed the feasibility of EGFR p.T790M prediction using deep genetic profiling of baseline tissue samples. The main finding was that subclonal features, such as the MATH score, subclonal CNV, as well as the ploidy, TMB, and SBS1 + 5 mutational signature, were significantly associated with the subsequent development of *EGFR* p.T790M. Of note, clinical and basic molecular characteristics, like age, smoking status, the type of *EGFR* mutation, and the presence of *TP53* mutations, showed no association with *EGFR* p.T790M in the study cohort, which could in part be due to the relatively small number of patients, but nevertheless underlines the superior performance of complex genetic biomarkers in this setting.

Previous studies have identified *EGFR* exon 19 deletions (up to 75%) and *TP53* mutations as significant discriminators between T790Mneg and T790Mpos patients [18], but only numerical differences without statistical significance were noted in our cohort (Table 1). In contrast to other reports that have focused on the characteristics of T790Mneg tumors, such as whole genome doubling [18], to discern them from T790Mpos cases, our study suggests that properties of the tumors that eventually develop the T790M resistance mutation are more important at baseline (before TKI treatment); all significant changes, such as higher MATH score, subclonal CNV, ploidy, TMB, and SBS1 + 5 mutational signature, point to either increased intratumoral heterogeneity and/or increased activity of mutational processes as a cardinal biologic characteristic of tumors poised to develop EGFR p.T790M. Of note, we could not reproduce the finding of lower TMB for T790Mpos cases in a previous study [18], as TMB was significantly higher in T790Mpos patients in our cohort. While the reasons for this discrepancy are unclear, the TMB estimation based on WES, as performed in the current study, can generally be considered more accurate than the panel NGS‐based TMB estimation employed in previous reports [32].

Another distinguishing characteristic of our study is that it focused on baseline tumor samples instead of samples collected at the time of disease progression, which may provide special opportunities for biological insights and clinical exploitation. For example, recalculation of the MATH score at the time of TKI failure from previously published data [18] showed no difference between T790Mpos and T790Mneg groups with uniformly high and similar values (0.35 versus 0.33). In contrast, MATH scores in our study at baseline were lower and dissimilar (0.29 versus 0.23, *p* = 0.03). This indicates an increase and convergence of the score with tumor progression, which can obscure the differences present at baseline. From a practical viewpoint, an important advantage of testing at baseline before TKI therapy is the potential ability to predict the subsequent development of *EGFR* p.T790M even before the first‐line treatment, which could be based on a combination of associated biomarkers. It should also be noted that the scarce presence of T790Mpos reads at baseline does not have any predictive value itself, as these have very low allelic frequencies and are equally present in the T790Mpos and T790Mneg groups. The most plausible explanation for their presence is C>T transition artifacts induced by formalin fixation [33]. One important implication of these observations is that sequencing of *EGFR* itself at baseline is insufficient for the prediction of the subsequent development of *EGFR* p.T790M.

Histopathological evaluation did not reveal a strong discriminator between different resistance mechanisms. Only the level of immune infiltration reached significance by single hypothesis testing. Immune infiltration appeared to be increased in T790Mpos cases in our cohort, which stands in contrast to previously published NGS‐based immune infiltration estimates where increased immune infiltration was observed for T790Mneg cases [18, 34]. Our results could be influenced by the small cohort size or the difference in study design as our samples were taken before the development of the resistance mechanism.

The main limitations of our study are the retrospective design and the relatively small cohort size, which precluded the development of a useful forecasting model. In addition, the high drop‐out rate raises questions about the technical feasibility of WES on a broad scale. However, it should be noted here that most dropouts in our study were due to the lack of tumor or normal tissue or due to the previous use of the scant tumor tissue available from small biopsies for earlier scientific projects (27 patients, 34%). Only a small number of cases (13 patients, 16%) could not be used due to library quality and DNA quantity. In fact, WES requires only a small amount of approximately 100 ng DNA, similar to that needed for panel‐based TMB estimation [35, 36] or comprehensive genomic profiling through large capture‐based panels of 400–500 genes, whose feasibility in the routine setting has already been demonstrated in several pivotal academic studies, like those involving MSK‐IMPACT [37]. Future studies could also expand the scope beyond genetic features to additionally include immunologic characteristics, whose particular characteristics and role in pathogenesis and treatment of EGFR‐mutated lung cancer are increasingly recognized [38].

In conclusion, this pilot study indicates an association between measures of tumor subclonality observed in the extended molecular profiling using WES at initial diagnosis and the subsequent development of the T790M resistance mutation in NSCLC patients receiving the first‐/second‐generation TKIs. Larger prospective studies are needed to validate these findings and accurately implement a forecasting model.

## Author contributions statement

MM, JB, AS and PC involved in conceptualization; MM, MK, DK, TKYT, MA, CA, JS, A‐LV, HG, MT, AS and PC contributed to data curation; MM, MK, KK, JB, AS and PC carried out formal analysis; AS and PC contributed to funding acquisition; MM, MK, MA, JS, CA, JB, AS and PC investigated the study; MM, JB and PC contributed to methodology; JB, AS and PC contributed to project administration; MT, JB, AS and PC contributed to resources; MM and JB helped for software; JB, AS and PC supervised the study; MM and PC validated the study; MM contributed to visualization; MM and PC contributed to writing – original draft; all authors contributed to writing – review and editing.

## Supporting information


**Figure S1.** Relative effect of complex genetic biomarkers on patient survival
**Table S1.** Characteristics of all study candidates
**Table S2.** Overview of mutations, tumor cell content estimation, *TP53* mutation status, SBS signatures, and histopathological evaluation
**Table S3.** Other mutations (besides *TP53*) detected in at least three cases in each of the study groups
**Table S4.** Recalculation of the MATH score in the samples from the study analyzing *EGFR‐*mutated lung cancers at the time of EGFR inhibitor failure
**Table S5.** Individual gene CNVs detected in the study samples
**Table S6.** LOH detected in the study samples
**Table S7.** Pairwise correlations between biomarkers in the study samples

## Data Availability

The data that support the findings of this study are available from the corresponding author upon reasonable request.
