# Supplementary material for: Genomic heterogeneity at baseline is associated with T790M resistance mutations in EGFR‐mutated lung cancer treated with the first‐/second‐generation tyrosine kinase inhibitors
Source: J Pathol Clin Res. 2024 Jan 17;10(2):e354. doi: 10.1002/cjp2.354 (PMC10792701; doi:10.1002/cjp2.354)
Supplement: Supplementary file 1 — Figure S1. Relative effect of complex genetic biomarkers on patient survival Table S1. Characteristics of all study candidates Table S2. Overview of mutations, tumor cell content estimation, TP53 mutation status, SBS signatures, and histopathological evaluation Table S3. Other mutations (besides TP53) detected in at least three cases in each of the study groups Table S4. Recalculation of the MATH score in the samples from the study analyzing EGFR‐mutated lung cancers at the time of EGFR inhibitor failure Table S5. Individual gene CNVs detected in the study samples Table S6. LOH detected in the study samples Table S7. Pairwise correlations between biomarkers in the study samples [file CJP2-10-e354-s001.zip › cjp2354-sup-FigureS1,TablesS1,S3,S4,S7.pdf]

Genomic heterogeneity at baseline is associated with T790M resistance mutations in *EGFR*-mutated lung cancer treated with the first-/second-generation tyrosine kinase inhibitors

M Menzel *et al*, *J Pathol Clin Res*, https://doi.org/10.1002/cjp2.354

Supplementary Figure S1
Supplementary Tables S1–S7 (for supplementary Tables S2, S5 and S6, see separate Excel files)

A

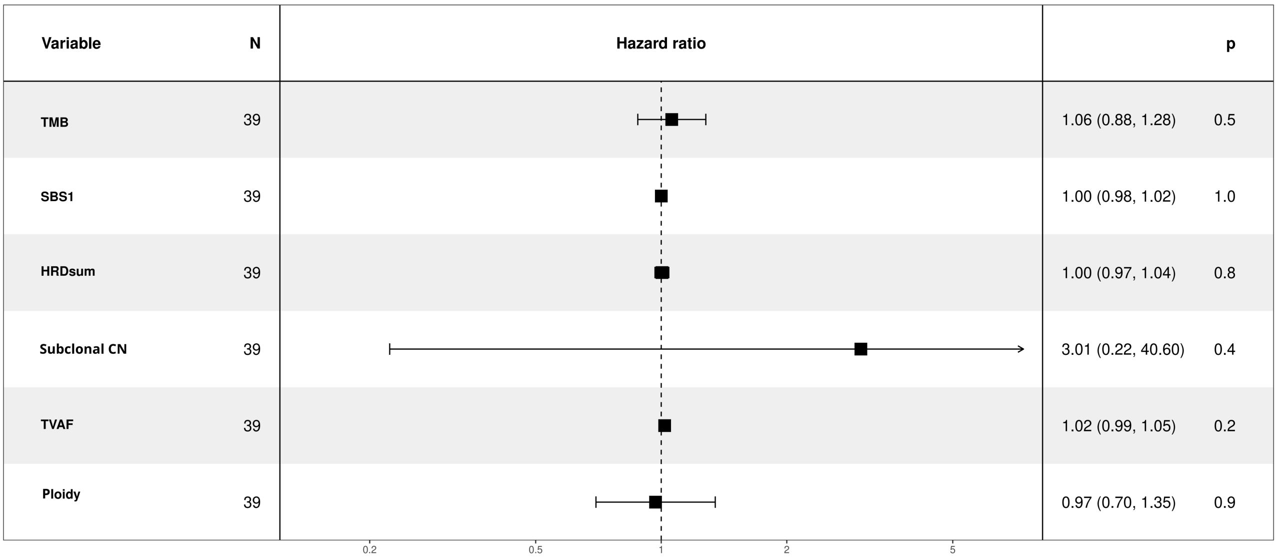

B

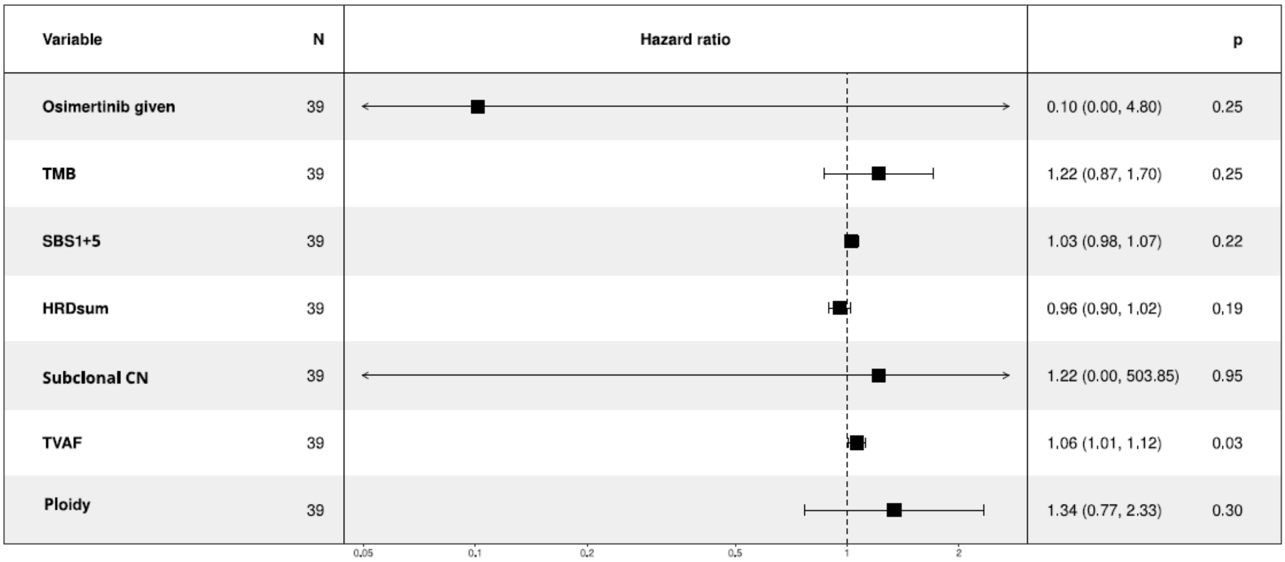

Figure S1. Relative effect of complex genetic biomarkers on patient survival.
The relationship between the complex biomarkers identified in this study and (A) progression-free, or (B) overall survival was analyzed using Cox regression.

Table S1. Characteristics of all study candidates

Among 79 study candidates, 39 patients could undergo whole exome sequencing (WES) of tumor and normal tissue and were included in this study (please see Table 1 of the main manuscript).

**All study patients**	**All patients** (*n* = 79)	**T790Mpos** (*n* = 51)	**T790Mneg** (*n* = 28)	*p* value
Age, median (IQR)	66 (57–75)	66 (57–75)	66 (60–76)	0.43
Sex, female, *n* (%)	56 (71%)	35 (69%)	21 (75%)	0.55
ECOG PS 0/1, *n*	42/36	28/22	14/14	0.61
Never/light smokers, *n* (%)	60 (76%)	39 (77%)	21 (75%)	0.88
Stage IV at initial diagnosis, *n* (%)	59 (75%)	36 (71%)	23 (82%)	0.26
Brain metastases, *n* (%)	17 (22%)	11 (22%)	6 (21%)	0.99
*EGFR*mut: del19/L858R/other, *n*	57/19/3	40/10/1	17/9/2	0.20
TP53 mutated/wild-type	16/23	12/13	9/5	0.25
PFS of 1L EGFR TKI, median (IQR), mo	13.6 (8.8–20.8)	14.4 (10.3–25.5)	10.2 (5.8–18.7)	0.63
OS, median (IQR)	33.7 (20.3–61.3)	38.0 (22.9–65.3)	23.1 (14.5–40.3)	0.045

ECOG PS: Eastern Cooperative Oncology Group performance status; n: number; 1L: first line; PFS: progression-free survival, OS: overall survival; mo: months.

Table S2. Overview of mutations, tumor cell content estimation, *TP53* mutation status, single-base-substitution signatures (SBS), and histopathological evaluation **(see separate Excel file)**

Table S3. Other mutations (besides *TP53*, for which please see Table S2) detected in at least 3 cases in each of the study groups. Statistical comparisons between the percentages of samples with each mutation in each group were performed with the Fisher's exact test.

**Gene**	**In T790Mpos**	**In T790Mneg**	**% yes**	**% no**	***p* value**
PARP4	5	0	20	0	0.14
RYR2	5	0	20	0	0.14
DGKK	4	0	16	0	0.28
FBRS	4	0	16	0	0.28
MUC19	4	0	16	0	0.28
MUC16	5	1	20	7	0.39
ABCA13	3	0	12	0	0.54
ANKRD11	3	0	12	0	0.54
APLP1	3	0	12	0	0.54
DNAH11	3	0	12	0	0.54
NRCAM	3	0	12	0	0.54
NRXN1	3	0	12	0	0.54
OR8H2	3	0	12	0	0.54
PAK2	3	0	12	0	0.54
PIK3CA	3	0	12	0	0.54
TPO	3	0	12	0	0.54
TP53	10	7	40	50	0.74
CSMD3	3	1	12	7	1
DCHS2	5	2	20	14	1
KLRC3	3	1	12	7	1
PRAMEF2	3	1	12	7	1
TTN	5	2	20	14	1

Table S4. Recalculation of the MATH score in the samples from study analyzing *EGFR*-mutated lung cancers at the time of EGFR inhibitor failure (published in PMID 34261696).

Case	MATH	Status
A038	0.457932142541407	Other
A040	0.375004375004375	Other
A048	0.383000403745218	Other
A056	0.335382570407909	Other
A057	0.217841971112999	Other
A058	0.226687231414888	Other
A083	0.377063918005154	Other
A089	0.254637644931226	Other
A092	0.322201017393943	Other
A097	0.285113479475274	Other
A256	0.336843640993674	Other
A436	0.452211890265947	Other
A440	0.370236814727302	Other
A441	0.371089819102715	Other
A450	0.276095131619067	Other
A456	0.252593060043793	Other
A465	0.354729658880719	Other
A466	0.373625714285714	Other
A472	0.402518399092361	Other
A477	0.494083736685494	Other
A478	0.318545667590896	Other
A003	0.643323425720565	T790M
A018	0.156338856411498	T790M
A032	0.322601799674916	T790M
A041	0.173149985540919	T790M
A042	0.477716095327041	T790M
A044	0.306712095400341	T790M
A047	0.406672551246839	T790M
A055	0.451924104566408	T790M
A060	0.508742113191912	T790M
A079	0.33462392471889	T790M
A085	0.298985965993445	T790M
A087	0.307205779663748	T790M
A093	0.191816264743134	T790M
A096	0.306327203107454	T790M
A138	0.361900588648071	T790M
A151	0.456900473205688	T790M
A152	0.30233	T790M
A180	0.295448683377494	T790M
A244	0.245452986061542	T790M
A317	0.384617633131252	T790M
A438	0.274725423499349	T790M
A442	0.342548732523756	T790M
A443	0.374647725587204	T790M
A448	0.411703016979238	T790M
A449	0.503031943026531	T790M
A451	0.231168831168831	T790M
A452	0.277777777777778	T790M
A453	0.311718378777309	T790M
A455	0.463335115833779	T790M
A457	0.215384736094656	T790M
A458	0.296942534119701	T790M
A459	0.325228954961736	T790M
A463	0.384790580651527	T790M
A464	0.432040400894337	T790M
A468	0.266544324678852	T790M
A469	0.526263952724885	T790M
A471	0.359695512820513	T790M
A475	0.39680706347373	T790M

Table S5. Individual gene copy number variations (CNVs) detected in the study samples **(see separate Excel file)**

Table S6. Losses-of-heterozygosity (LOH) detected in the study samples **(see separate Excel file)**

Table S7. Pairwise correlations between biomarkers in the study samples (Pearson's R).

Metric 1	Metric 2	Correlation
CNA count	subclonal TMB	−0.108641393196919
CNA count	TMB	−0.101630674027896
MATH	subclonal TMB	−0.0530329543729697
ploidy	TVAF	−0.0355607595844036
CNA count	TVAF	−0.0334746861223699
subclonal TMB	fraction_subclonal_cn	−0.0289840388824905
ploidy	subclonal TMB	0.0279867115507999
MATH	TMB	0.0390250333208036
TVAF	fraction_subclonal_cn	0.0442250173860612
CNA count	fraction_subclonal_cn	0.0442653352888444
HRDsum	CNA count	0.0699886391110559
fraction_subclonal_cn	TMB	0.11221924317924
ploidy	TMB	0.118283596691111
CNA count	MATH	0.145037968720855
SBS1+5	fraction_subclonal_cn	0.162490036551618
ploidy	CNA count	0.179423789981054
CNA count	SBS1+5	0.18588816293507
SBS1+5	TVAF	0.20601629743894
TVAF	TMB	0.226418022976519
HRDsum	fraction_subclonal_cn	0.289745899501273
SBS1+5	MATH	0.315405491721007
MATH	fraction_subclonal_cn	0.328309143228979
TVAF	MATH	0.329292640227317
HRDsum	TMB	0.338472455381114
SBS1+5	TMB	0.341915400653456
ploidy	MATH	0.36753251014295
SBS1+5	subclonal TMB	0.370519837684927
ploidy	HRDsum	0.391778366918732
TVAF	subclonal TMB	0.42307912748877
HRDsum	subclonal TMB	0.42598653069864
ploidy	fraction_subclonal_cn	0.434593076717595
HRDsum	MATH	0.467789922627037
ploidy	SBS1+5	0.505046253691821
HRDsum	TVAF	0.55973362625915
HRDsum	SBS1+5	0.679537471273204
subclonal TMB	TMB	0.79314886223022
